# Evaluation of the impact of patient and public involvement on doctoral students in palliative dementia care research

**DOI:** 10.1186/s40900-025-00715-1

**Published:** 2025-07-03

**Authors:** Woo Suk Yang, Joshua Rothwell, Matthew Severyn, India Tunnard, Toslima Khatun, Jane Ward, Nathan Davies, Charlotte Kenten, Elizabeth L. Sampson, Catherine J. Evans

**Affiliations:** 1 Cicely Saunders Institute of Palliative Care, Policy & Rehabilitation, Florence Nightingale, Faculty of Nursing, Midwifery & Palliative Care, King’s College London, London, UK; 2 Marie Curie Palliative Care Research Department, Department of Psychiatry, University College London, London, UK; 3 GKT School of Medical Education, Faculty of Life Sciences and Medicine, King’s College London, London, UK; 4 Centre for Psychiatry and Mental Health, Queen Mary, Wolfson Institute of Population Health, University of London, London, UK; 5 Research Department of Primary Care and Population Health, University College London, London, UK; 6 Department of Psychological Medicine, Royal London Hospital, East London Foundation Trust, London, UK; 7 Sussex Community NHS Foundation Trust, Brighton, East Sussex, UK

**Keywords:** Public involvement, Dementia, Doctoral research, Qualitative research, Documentary analysis

## Abstract

**Background:**

Patient and Public Involvement (PPI) has moral and practical importance by empowering stakeholders to influence research. Research by doctoral students is a major component of accepted research and research training, yet the literature on meaningful PPI in this area is limited. We aimed to provide insights into the impact of PPI in doctoral research and education, and integrate the findings with identified published evidence to develop the Empowering Better End of Life Dementia Care (EMBED-Care) Guideline for Reporting and Evaluating PPI in doctoral research for future evaluations.

**Methods:**

Documentary analysis was undertaken of the PPI panel meeting notes and written reflections by doctoral students from the five doctoral projects in the EMBED-Care programme. The data extracted were analysed using a deductive thematic approach guided by Staley’s INVOLVE framework comprising nine themes (research agenda, design and delivery, ethics, impact on public involved, researchers, participants, wider community, community organisations, wider change). To compare and interpret the findings, a literature review in PUBMED identified published evidence on PPI in doctoral research with final data integration to construct the guideline.

**Results:**

There was at least one impact of PPI identified in each of the nine respective themes with the most common being ‘impact on research design and delivery’. The identified impacts were universally described as positive for the doctoral students, their projects, PPI members and the wider community. Published studies (*n* = 21) reporting PPI in doctoral research echoed findings of positive benefits, but the methods to utilise PPI and the reporting on outcomes were inconsistent. The EMBED-Care Guideline for Reporting and Evaluating the Impact of PPI on doctoral research was constructed to standardise PPI reports and information collected, and reflection on the impact and outcomes, to facilitate evaluation of impact in future research.

**Conclusions:**

PPI primarily benefited research design and delivery, but had a variety of social benefits to the researchers, public members, study participants and wider communities. The findings demonstrate the vital role of PPI in the academic development of doctoral students to enhance skills and expertise, and research design and delivery.

**Supplementary Information:**

The online version contains supplementary material available at 10.1186/s40900-025-00715-1.

## Abstract (plain language summary)

Patient and public involvement (PPI) is the practice of working with members of the public to improve public services and research. Doctoral students are researchers in training. This article investigates the impact of PPI on five doctoral students in the Empowering Better End of Life Dementia Care (EMBED-Care) programme, to reach a better understanding of how PPI can be used to benefit doctoral research and education. The findings informed the development of the EMBED-Care Guideline for Reporting and Evaluating PPI in doctoral research.

Analysis of PPI panel meeting notes and written reflections of the doctoral students on impact of PPI on their respective studies showed that PPI has benefits for the research and those that take part in the process, including the doctoral students and the public members. The most common benefit of PPI was its impact on research design and delivery. There were a variety of other impacts, including enjoyment for PPI members and research expertise and personal development of the doctoral students.

The findings aligned with the published evidence on PPI in doctoral research and corroborates how PPI is vital in doctoral research and education. However, across the published studies on the use of PPI in doctoral education, the methods of data collection were inconsistent and varied in detail. The EMBED-Care Guideline for Reporting and Evaluating PPI in doctoral research was created from the study findings to support future researchers in capturing relevant information and encouraging detailed reflections to evaluate impact and drive improvements continually.

**Supplementary Information:**

The online version contains supplementary material available at 10.1186/s40900-025-00715-1.

## Background

### What is the role of patient and public involvement?

Patient and public involvement (PPI) refers to research that is conducted in collaboration with members of the public rather than being conducted on, about, or for them, and focuses on PPI members as ‘lived-experts’ [1, 2]. This practice mirrors the shift from a paternalistic to a patient-focused model of healthcare delivery, which emphasises the importance of public opinion and participation [3–7]. Researchers obtain input from the end-beneficiaries of public institutions, such as people receiving healthcare in the National Health Service (NHS), to optimise the provision of a service [8–11].

PPI is important to enable effective and relevant research and public services. Moreover, it has a moral significance in ensuring that the public who are the funders and end-beneficiaries have an active role in the development and implementation of research and services [3]. Assessments into the impact and benefits of PPI have been conducted since the 1990s [2]. This evidence base identifies a breadth of benefit from PPI, including empowering public members, building rapport and partnerships with communities, increasing representation of underserved groups to mitigate health inequities and improving and demystifying the research process to enable research participation and develop researchers’ knowledge about the area of study [12–14]. PPI serves an important role as a bridge between researchers and people affected by disease. This is particularly important for underrepresented groups in research, such as those with advanced dementia [12].

However, the variability of impact, benefit, outcomes, and the diversity of applications of PPI make it difficult to generalise the evidence base onto a specific scenario, such as doctoral research. Study at doctoral level is the highest form of academic training for researchers. It is a vital foundation to pursue a career as an independent researcher, leading research studies. There are increasing numbers of doctoral students worldwide, for example, in 2020, there were around 55,000 doctoral students in the United States (US) and 21,000 in the United Kingdom (UK) [15, 16]. Despite this being the highest level of research training, the published evidence on the impact and benefit of PPI in doctoral research is limited [12, 13]. Further investigation is required to understand the value and impact of PPI on doctoral research studies and training.

### Why is doctoral research in palliative dementia care important?

Improving the quality of education of the next generation of researchers in palliative dementia care is vital to build capacity and tackle the global challenge of meeting rising needs for services and care [17]. Dementia is an umbrella term for progressive terminal neurological conditions with profound cognitive decline that compromises all areas of function with disease progression, with Alzheimer’s disease as the most common type of dementia [18]. Dementia is the leading cause of death in England and Wales [19]. The prevalence of dementia is growing rapidly, associated with increasing longevity. This is projected to continue with the number of people with dementia projected to reach 1.7 million by 2040 [20]. Similarly, globally, the number of dementia cases is expected to rise from 57.4 million in 2019 to 152.8 million by 2050 [21]. This trend is accompanied by a corresponding increase in demand for healthcare, and the economic societal cost is estimated to rise from $2.8 trillion in 2019 to $16.9 trillion in 2050 [22].

### What is the context for doctoral research on palliative dementia care?

This study draws upon a major programme of research on palliative dementia care called Empowering Better End of Life Dementia Care (EMBED-Care). The EMBED-Care programme is the largest funded study on palliative dementia care in the UK. The programme is a collaborative effort involving clinicians, researchers, policymakers, patients, and their families, which aims to develop interventions to address the priorities of individuals severely affected by dementia [23]. Capacity building in palliative dementia care and PPI were major components of the programme to nurture future research leaders in this field and innovate public involvement in dementia research. The programme incorporated five doctoral projects concerning:

A. Shared decision-making for people with dementia living at home, family carers and practitioners.
B. Implementation of an eHealth intervention to optimise person-centred assessment and decision-making for people with dementia in care homes.
C. Patterns of healthcare use and quality of care among people with dementia nearing the end of life using routine data.
D. Grief, burden and the role of social and professional support in family carers of people living with dementia.
E. The palliative care needs of people living with Frontotemporal Dementia.

### Aim

The study aimed to evaluate the impact of PPI on the doctoral projects within the EMBED-Care Programme and the role of PPI in doctoral research and education. The findings informed the development of the EMBED-Care Guideline for Reporting and Evaluating PPI in doctoral studies.

## Methods

### Study design

The study followed a documentary analysis approach using deductive thematic analysis [24] to evaluate the impact of PPI on the doctoral research studies and training. The study was undertaken within the EMBED-Care programme as part of the ongoing process to evaluate PPI. The intention was to drive continual improvements in the approach aligned with the UK National Standards for Public Involvement in research, standard six on PPI impact to ‘Seek improvement by identifying and sharing the difference that public involvement makes to research’ [1]. Reporting was informed by the Guidance for Reporting Involvement of Patients and the Public 2 (GRIPP2) [26] as a standardised structure to provide clarity on the aim, methods, results, impact and reflections of PPI (Additional file 1: GRIPP2 Short Form).

The study was underpinned by Staley’s INVOLVE report [25] detailing nine themes for PPI impact on research. These themes were considered robust, generated from an extensive analysis of PPI impact on research that encompassed a wide variety of PPI studies, going beyond doctoral research [25]. Staley’s nine themes comprised:

1. **Impact on the research agenda** - impact on identifying topics for research, shaping research questions, initiating research projects and making decisions about which projects to fund.
2. **Impact on research design and delivery** - the project design, the research tools and methods, recruitment, data collection, the analysis of data and writing and dissemination.
3. **Impact on research ethics** - improving the consent process and helping researchers to develop ethically acceptable research.
4. **Impact on the public involved** - the positive benefits include acquiring new skills and knowledge, personal development, support and friendship, enjoyment and satisfaction and being rewarded financially. The negatives include being emotionally burdened, overloaded with work, exposed through the media and frustrated at the limitations of involvement.
5. **Impact on researchers** - the positive benefits include a better knowledge and understanding of the community, enjoyment and satisfaction, career benefits and challenges to beliefs and attitudes. The reports of negative impact include an increased demand on resources and a slower pace of research, loss of power, forced changes in working practice and challenges to values and assumptions.
6. **Impact on research participants** - a better research process, helping people to feel more at ease in interviews, providing emotional support, providing access to information and services and offering hope and inspiration.
7. **Impact on the wider community** - create trust and acceptance of the research, keep projects grounded and focused on benefits for the community and improve relationships between the community and professionals.
8. **Impact on community organisations** - gain credibility for the other activities they work on, gain credibility as community leaders, increase their knowledge and understanding of a health or social condition, gain public recognition through disseminating the information to the community and participating in conferences, make a positive contribution that benefits the community, become a link between the mainstream health system and people who want or need to use services and develop new alliances which has furthered their ability to influence the research agenda.
9. **Impact on implementation/change** - bring about change, particularly in developing new services or improving existing ones.

### Data acquisition and eligibility

The data consisted of the written reflections by doctoral students and meeting notes from the PPI Study Reference Panel for the EMBED-Care programme. The PPI panel consisted of public members with lived experience of dementia as bereaved family carers (*n* = 6), a current family carer of a person living with dementia (*n* = 1) and people living with dementia (*n* = 2). The panel was chaired by a former family carer (JW) whose role was supported by senior researchers (lead researchers for PPI in the EMBED-Care programme ND/CE) and coordinated by the programme manager (CK) and, a researcher (IT). Most public members had previous experience of PPI in research, and several were involved with charitable organisations campaigning for excellent dementia care for all. The PPI members and doctoral students were informed of the study and provided verbal agreement for analysis of the meeting notes and written reflections. Ethical approval for the wider EMBED-Care programme from the London Queen Square Ethics Committee and Health Research Authority (HRA) on 01.06.2022 (Ref20/LO0295).

The doctoral students consulted with the PPI panel about their doctoral projects bi-annually across the duration of their respective doctoral study. The data reports PPI panel meetings with doctoral students from January 2020 to November 2021. This encompassed the first one to two years of doctoral study to inform and improve the process of the PPI panel working with the doctoral students. The written reflections were intended to support each student in developing their expertise in PPI in research and support the continual evaluation of the PPI approach within the wider research programme. The written reflections were completed by the doctoral student after each PPI meeting summarising what was asked of the PPI members, the responses of the PPI members and reflection on the impact of the meeting on their research.

### Data analysis

Data analysis included three stages: (1) Documentary analysis of PPI panel meeting notes and student written reflections; (2) Interpretation of the data in relation to the published evidence on PPI in doctoral studies; and (3) Constructing the guideline for reporting and evaluating PPI in doctoral research.

#### Documentary analysis of written reflections and fieldnotes

Data analysis used codebook thematic analysis informed by Braun and Clarke [24]. The nine themes on PPI impact from the Staley INVOLVE report formed the initial codebook [25]. The codebook was used to develop a bespoke data extraction sheet in Google Sheets [27]. One researcher (WY) familiarised themselves with the data and extracted the relevant data detailing impact on the research into a spreadsheet. Each category formed a code for the respective type of impact. Three researchers (WY, JR, and MS) then read the Staley report [25] to familiarise themselves with the themes and their definitions. Each researcher then independently coded the extracted data by the respective impact code. Where there were differences in interpretations of the data, the researchers discussed the data further to reach a consensus. The process was overseen by CE, an experienced qualitative researcher with expertise in public involvement in research.

#### Data interpretation in relation to the published evidence

To interpret the findings, a literature review was undertaken on published studies evaluating the impact of PPI in doctoral research. The intention was to compare the findings with the published evidence to construct the Guideline for Reflecting on and Evaluating PPI in doctoral research. We identified studies by searching the PUBMED database for publications with “Patient and public involvement” AND “Doctoral”, OR “Public and patient involvement” AND “Doctoral” in the title or abstract. The study eligibility criteria comprised: concerned PPI in doctoral research, a primary research study (quantitative, qualitative or mixed-methods), peer-reviewed, and published and available as a full-text manuscript in English. Screening and identification were undertaken by one researcher (WY).

#### Constructing the guideline for reporting and evaluating PPI in doctoral research

The findings from the documentary analysis, and convergence, corroboration and divergence with the published evidence informed the construction of the guideline for reporting and evaluating PPI in doctoral research. This enabled identification of key areas to facilitate standardisation of reporting in PPI panel meeting notes and written reflections on the impact of PPI on the researcher and the research.

## Results and discussion

### PPI panel meetings with doctoral students

Each of the five doctoral students met individually with the PPI panel 2–3 times during the study reporting period over 23 months, totalling 11 PPI meetings on the doctoral studies for the EMBED-Care programme. Meeting length ranged from 45 to 75 min. Student written reflections and meeting notes were available for each meeting.

The impact of PPI on the doctoral students was universally positive. Table 1 details what was asked of the PPI members in each meeting and the impact of the discussion on the research and researcher, presented as themes using the Staley INVOLVE framework. The impacts encompassed a diversity of themes for the researchers and the wider community. The impacts mostly concerned improving the respective research study design and delivery, for example using more open questions during interviews and improving the wording of text to foster rapport with participants. One doctoral student described how a PPI meeting had an important role on the research process.

*I subsequently wrote about the limitations of the routine data in my upgrade/thesis section. Their feedback around quality indicators (population vs. individual experiences and carers) were also embedded into the published paper’s discussion section.* [doctoral student 5]

Yet, there was also marked diversity of benefit. The findings showed how the impact of PPI exceeded beyond the core features of the research project to have and improve on their personal development. For example, one doctoral student described how their research skills developed in working with the PPI panel:

*It allowed me to practise disseminating my research to a PPI audience. This can be particularly challenging in my area of research as palliative*,* and end of life care can be a sensitive topic.* [doctoral student 4]

**Table 1:** Impact of patient and public involvement (PPI) in doctoral projects mapped as themes using Staley’s INVOLVE framework [25]

PPI panel meeting focus by doctoral project	Illustrative extracts from student written reflections	Theme and description of the PPI impact by doctoral project
**Project A** **Meeting 1** • What does shared decision-making look like for people with dementia at home and their families • Using the outcome measure to enhance comprehensive assessment and shared decision-making **Meeting 2** • Outcomes to consider for family carers as a result of enhanced shared decision-making • How is the term ‘training’ received? Is there an alternative?	The discussion with the PPI directly impacted aspects of the intervention development in home care. For example, in terms of how different priorities of carers and patients may be managed. All the discussion points were taken to the co-design workshop and helped to better understand how the EMBED-Care intervention will be used as well as what is required to support use. Outcomes being considered for the feasibility study includes social services and carer burden. Training was discussed at length in the co-design workshop to support wider use of the intervention	**Research design and delivery**: improved understanding on how the intervention is used in the study. **Wider community**: ensuring that the intervention is grounded in the priorities for carers and patients. **Implementation/change**: the discussion informed ways to strengthen the intervention and build the capacity for eventual change in healthcare. **Research agenda**: the PPI group played an important role in determining the research direction through deciding which outcomes to focus on for the study. **Research design and delivery**: improved by the PPI group’s role in identifying the appropriate outcomes for the study and the language used when discussing with carers. **Research participants**: The PPI group’s recommendation to use different terms for family carers enhanced ways of working with participants **Wider community**: positive impact by keeping the project grounded in the needs of carers and improving relationships with researchers.
**Project B** **Meeting 1** • Using digital health app in a care home– what do we need to consider for a person with dementia? **Meeting 2** How the EMBED-Care intervention/ assessment could be utilized in care homes– family carer preferences	Has fed into design and development of the app and the intervention Informed development of the co-design workshops content. Took these ideas into discussions in the groups. PPI members agree with all points. Has been good to get to know the students and feels their confidence in talking to PPI group has built which will support working with the public. This discussion fed directly into further app development and the workflow of the intervention in terms of how alerts should be used and family members involved It also fed into further co-design discussions in which plans were refined	**Research design and delivery**: improvements to the intervention for the context of people with dementia in care homes and using an app for the intervention delivery. Further development of the content for the co-design workshops and refinement of the research plan. **Public involved**: the public members report enjoyment of working with the PhD students and that process will support how the researchers work with the public in their research. **Researchers**: improved confidence in presenting and talking to the PPI group and working with the public. **Wider community**. consideration of the role of the family helped to ground the research in the context of the community
**Project C** **Meeting 1**: Presented three things– i. What is big data? Some information around terminology and how it relates to research with people with dementia ii. Preliminary findings from the systematic review of quality indicators iii. Preliminary findings from the descriptive study **Meeting 2**: Presenting the findings from the descriptive study focusing on the patterns of unplanned hospital admissions of people with dementia from diagnosis to the end of life.	“Their feedback into the way I presented routine data was helpful in subsequent dissemination of the work.” [doctoral student] “For the third component of my PhD, I made efforts to include any possible variable related to carers’ involvement in care of people with dementia into my data extraction and data analysis.” [doctoral student] “This was very recent, but it definitely helped with the interpretation of findings and possibly for some sensitivity analyses for my work” [doctoral student]	**Research design and delivery**: improved consideration of the limitations of the study design in the reporting, enhanced the data analysis plan with inclusion of variable on carers involvement and the dissemination plan. **Wider community**: detailed consideration in the data analysis of the support provided by carers strengthening the grounding of the research for the wider context. **Research design and delivery**: improved through support in the interpretation of the findings in relation to people affected by dementia and considerations for the sensitivity analysis.
**Project D** **Meeting 1** • Introduced the role of trust and confidence in how effective support is for people- both from services and family and friends • Explored initial ideas from the systematic review findings and how these may lead into research questions and a theory of grief in this population **Meeting 2** • Social support, what does feeling supported mean in the context of being a family carer for a person with dementia, and what needs to happen for support to be helpful?	“I took the idea of trust and confidence to the wider PPI group and then included it as a question in my qualitative interviews.” [doctoral student] Included open text questions in carer interviews about social support from services and from peers. “Reinforced that the links I had made from my review and prior experiences were relevant and relatable (which was particularly helpful after spending lots of time being immersed in the literature but less involvement with actually talking to carers)” [doctoral student] Ensured that the quantitative measures and qualitative interviews capture negative or unhelpful experiences also.	**Research design and delivery**: changes to the topic guide with inclusion of themes on trust and confidence. **Research participants**: exploring the definitions of trust and confidence among dementia carers strengthened the research process. **Wider community**: discussing trust and confidence enhanced the grounding of the research within the community.
**Project E** **Meeting 1** • Meeting with public members to discuss my study protocol and ethics application for the cohort study **Meeting 2** • Exploring the concept of ‘total pain’ and its suitability to symptoms of [dementia type] **Meeting 3** • Updated the group on the cohort study and asked the group about what type of change they would like to see in the world of dementia care. • Reviewed baseline demographics from the cohort study. • Discussion around need for the research.	“This meeting strengthened my protocol as I mentioned having PPI input in my ethics application.” [doctoral student] “It allowed me the opportunity to showcase my work with other professionals in the sector using the regional facilitator meeting and simultaneously advertise my study. It helped guide my project and ensure that the needs of people with [dementia type] were being heard at a PPI level.” [doctoral student] “This has helped steer my PhD as I now know the importance of publicly engaging with my research. Not just as part of EMBED-Care but also disseminating my own PhD.” [doctoral student] “It [working with PPI members] has allowed me to think about the future of this research and the need for further evidence.” [doctoral student]	**Ethical research**: This meeting directly benefited the process of developing the ethical application. **Researchers**: developed ability to disseminate research to a PPI audience and to showcase their work and to advertise their study. **Wider Community**: opportunity to pursue wider public engagement with community organisations. **Implementation/change**: develop capacity of community organisations for **Research design and delivery**: improved research dissemination plan **Researchers**: benefitted from improved knowledge and understanding on the experience of the dementia type, and developed their research interests and future direction. **Research agenda**: influenced the direction for future research. The researcher

### Interpreting the findings with the published evidence

The literature search yielded 34 papers. Twenty-one studies met eligibility of reporting PPI impact on doctoral research [28–48]. Additional file 2 summarises the 21 studies and impact on research. Comparing the findings with the 21 published studies showed a congruent pattern with the study findings. When utilising the themes from Staley’s INVOLVE report, most impact from the findings, and in the published studies, centred around improving research design and delivery. There was also a range of positive outcomes for the people involved that conveyed this was not a transactional process alone of a task, but also relational in building meaningful partnerships in working together [29–47]. Throughout the research process, the findings showed social and emotional benefits to researchers and the public members, illustrated in this quote:

*I’ve enjoyed sharing with you* [the researcher], *sharing information*,* thoughts*,* plans*,* all the things I’ve enjoyed. I’ve enjoyed caring about you and what’s happened to you*, [laugh] *and your disappointments*,* and the stress…* - comment by public member in a study on an exercise guide website for people with lung cancer by Curry et al. [31]

The benefits to the researchers included fostering their personal development as academics and providing insight into the perspectives of public members. This shows that PPI has the potential to enrich the doctoral educational experience beyond improving research projects. PPI holds an important role in connecting researchers to the public as representatives of the intended end-beneficiaries. This approach potentially ensures that doctoral programmes can develop academics that pursue research grounded in the lived experience of public stakeholders.

### Wider social benefits of PPI

PPI members agreed that the meetings were a great opportunity to connect with people while contributing to meaningful research. The findings in Table 1 reflect this. Across the doctoral studies the impact of PPI is demonstrated on researchers, participants, PPI members and the wider community. The findings resonate with the published studies of the profound emotional benefits reported by members [49–55]. Promoting the benefit of PPI of providing a sense of community and a fulfilling activity could improve the recruitment of public members in research involvement. This is predicated on meaningful involvement in research and inclusive approaches for involvement [56].

Members of the EMBED-Care PPI panel reported two primary social benefits of PPI. The first centred around the feeling of being able to help mentor a doctoral student by sharing personal experiences. The second was specific to the construction of PPI groups, where members benefitted from meeting others with shared experiences, and sharing their perspectives and connections to dementia. During a PPI meeting based on developing an eHealth intervention to optimise person-centred assessment and decision-making, one of the PPI members commented on their positive experience.

*I liked learning more about what they’re doing and contributing to their work. Would like the option to meet with them again to see how our contributions have helped and how their work is progressing*. [PPI member 1]

Beyond the researchers and public members involved directly in the PPI meetings, PPI had important further impacts on research participants, the wider community and society as a whole. For example, one PPI meeting discussing the terminology used for carers involved in an intervention resulted in a change to language for participants to feel comfortable. This also ensured that the research remained grounded within the community and the needs of its members.

*We’ve changed the term ‘training’ to ‘coaching’ for family carers as it sounds more supportive. I will explore this in the co-design workshop in November too.* [doctoral student 1]

Wider society benefitted from PPI through improving adherence to ethical frameworks, facilitating networking and building the capacity for future change. In one meeting between a doctoral student and PPI member, the PPI member discussed the ethical application and offered to make an introduction with regional facilitator groups.

*This meeting strengthened my protocol as I mentioned having PPI input in my ethics application…It allowed me the opportunity to showcase my work with other professionals in the sector using the regional facilitator meeting and simultaneously advertise my study.* [doctoral student 4]

The wider social benefits were frequently corroborated within the published studies exploring the role of PPI in doctoral research. This was particularly present for patient groups that had unique needs, such as accessibility issues for people with cerebral palsy.

*Adaptations made this study accessible to people with cerebral palsy who had additional impairments* - comment by doctoral student from a study reflecting on the experiences collaborating with PPI members with cerebral palsy by Manikandan et al. [39]

### How PPI can benefit future doctoral research

Both the EMBED-Care programme and wider evidence indicated that the most impactful element of PPI was on the research design and delivery theme. This likely relates to this theme broadly encompassing all stages of the research process, such as, exploring findings from the systematic review to inform the qualitative interview topic guide (project B), designing the content of the co-design workshops (project C), and advocating for the needs of specific groups e.g. helping recognise symptoms for adults with young onset/rare dementias (project E). Improving accessibility of information and reaching specific groups was corroborated in the published evidence and, wider evidence of impact of PPI in more complex areas such as recruitment methods and gaining access to community networks, data interpretation and dissemination [57–62].

PPI also provided an opportunity to discuss ethical considerations and help ensure that participants were able to give an informed consent to research. In some cases, the input of the PPI panel had a direct benefit to ethical review of the project by being able to respond positively to the enquires of the ethical committee. This was particularly beneficial for doctoral students with often little prior experience of the ethical review process.

The students indicated how they had benefited from including PPI in the initial stages of developing and confirming the research question, advocating for PPI to be included as early as possible and continuing throughout the research journey (see Table 1). This enabled the students to develop their research focus and agenda. This consideration was echoed in the published studies, for example:

…*being aware of PPI sooner*,* would have changed the way we designed the app for breast cancer patients. It would have changed my own PhD project. For me*,* it was somewhat too late*,* however I am making sure that my students and my work colleagues are aware of PPI and if necessary*,* take it into consideration when designing new research projects*.– reflection from a doctoral cancer nurse researcher published in a study by Tanay et al. [44].

Evidence from this study and other similar studies showed that PPI has a positive impact on the development of doctoral researchers with very few negative outcomes. To improve the quality of learning from doctoral education, the PPI process should be implemented into their studies as early as possible and continued throughout to write-up and dissemination.

### Reporting and evaluating PPI in doctoral research

The NIHR UK Standards for Public Involvement states that it is good practice to create processes to help reflect on public involvement [1], and to act on the benefits resulting from public involvement [1]. Subsequently, where possible, it is recommended for PPI incorporation into doctoral research to ensure a higher quality of research output and training.

To strengthen meaningful and impactful PPI in doctoral studies, the study findings, and the published evidence, informed the development of the EMBED-Care Guideline for Reporting and Evaluating the Impact of PPI in doctoral research. The guideline intends to support the process of reflection on, and evaluation of, the impact of PPI in research and drive improvements in meaningful involvement (Additional file 3 details the guideline).

The guideline details standardised aspects of the information collected to support reflection and evaluation of the impact of PPI. This consistency in reflections and reporting intends to improve analysis and interpretation of the information gathered from, for example PPI panel meeting notes and student reflections. The guideline provides a section to record the context of the PPI meeting, including the subject of research, stage of research and objective(s) of the meeting. This contextual information would enable monitoring and evaluation of impact, and change over time. For example, in PPI panel meeting notes, recording the stage of the doctoral research journey (i.e. months from registration, planned submission date).

It is inevitable that doctoral studies in different stages and contexts will likely have a different distribution of PPI impacts. An example of variability between studies was the implementation of PPI at different stages of the research process. Some doctoral students involved themselves with PPI very early on in their research, sometimes even before they began. For the majority, however, the journey began after a research topic or question had been decided and when the methodology and methods were in development.

The guideline was designed to incorporate perspectives for all participants of a patient and public involvement meeting. This is to ensure that the opinion of all people present at the meeting is captured, increasing the richness and diversity of responses, and to minimise missing PPI impacts that were not recalled or recorded. Systematic enquiry is required to obtain a complete picture of the impact of PPI on research, from the multiple perspectives of the researchers and the public members, and over time to track and evaluate impacts across the research cycle from identifying the research question to dissemination.

Improving the audit trail and context of the data was key to understand and evaluate how, when, and why the impacts observed occurred. Improving the recording of impact within PPI processes may strengthen ways of working, such as increasing the inclusivity and diversity of the public members to reflect the population of focus within the study. Public members can vary widely in experience and background, and researchers in their expertise in public involvement and working in inclusive ways. To inform inclusivity and diversity at an early stage, and to monitor this over time, the guideline has a section focusing on the background of the individual completing it, for example, ethnicity, age group, gender, lived experience of the area of study. These characteristics may be anonymous to fulfil its primary purpose of understanding the diversity and inclusivity of the group, and to identify and address areas of underrepresentation relevant to the study.

For future PPI reflection, it is important to state all positive and negative outcomes. Most PPI research has reported overwhelmingly positive outcomes [28–48]. This evidence needs to be considered with caution, as it is necessary to ensure that public members and researchers feel empowered and enabled to report constructive negative comments where possible. This may be the case due to politeness and aversion to negativity. The guideline emphasises that negative impacts are important to detail as opportunities to strengthen how the researchers and public members work together to build trust and partnerships and enable a more accurate evaluation of PPI in research. EMBED-Care was a major research programme with an established PPI panel that was developed to support the doctoral students. However, for standalone doctoral projects, a separate PPI budget may be required to provide PPI infrastructure and support.

### Strengths and limitations

This study has many strengths in the quality and quantity of the data available to evaluate the impact of PPI on doctoral research. However, the methods to collect information for evaluation could have been more thorough and standardised across the doctoral projects. Furthermore, despite the congruence between the ways that PPI benefitted these doctoral projects and the published evidence, it is uncertain as to what extent these findings can be generalised beyond the environment present in this study. Utilising PPI within different healthcare systems, government structures, research funding requirements and outside of developed countries may significantly change the role of PPI within doctoral research.

## Conclusions

The EMBED-Care documentary analysis shows that PPI benefits researchers, public members, study participants and the wider community by forming meaningful social connections. These impacts support the use of PPI in doctoral education, as a tool to improve research design and as an opportunity to engage with the public and promote mutual learning. This provides benefit to the wider public by creating opportunities for fulfilling relationships in contributing to the development of the next generation of academics. Although there may be significant financial costs and administrative barriers, there is evidence from this study to suggest that PPI should be incorporated as early as possible in doctoral education and continued throughout the research project. For future doctoral research, further implementation of PPI in doctoral studies would likely benefit the educational experience, research quality, the PPI members involved and the wider community who will receive healthcare. In future research, using the EMBED-Care Guideline for Reporting and Evaluating PPI impact on research intends to support planning for, and consistency and completeness in, the information collected, and the analysis, to evaluate fully the impact of PPI and change over time.

## Electronic supplementary material

Below is the link to the electronic supplementary material.

Supplementary Material 1

## Data Availability

The datasets used and/or analysed during the current study are available from the corresponding author on reasonable request.

## Abbreviations

EMBED-Care: Empowering Better End of Life Dementia Care
ESRC: Economic and Social Research Council
GRIPP2: Guidance for Reporting Involvement of Patients and the Public 2
NHS: National Health Service
NICE: National Institute of Health and Care Excellence
NIHR: National Institute for Health Research
PPI: Patient and Public Involvement
PhD: Doctorate in Philosophy
UK: United Kingdom
US: United States
