# Supplementary material for: Evaluation of the impact of patient and public involvement on doctoral students in palliative dementia care research
Source: Res Involv Engagem. 2025 Jul 3;11:73. doi: 10.1186/s40900-025-00715-1 (PMC12224461; doi:10.1186/s40900-025-00715-1)
Supplement: Supplementary file 1 — Supplementary Material 1 [file 40900_2025_715_MOESM1_ESM.docx]

# Additional files

## **Additional file 1: GRIPP-2 Short Form**

| **Section and topic** | **Item** | **Page where it is reported** |
| --- | --- | --- |
| 1: Aim | Report the aim of PPI in the study | Page 8 |
| 2: Methods | Provide a clear description of the methods used for PPI in the study | Page 8-12 |
| 3: Study results | Outcomes—Report the results of PPI in the study, including both positive and negative outcomes | Page 12-22 |
| 4: Discussion and conclusions | Outcomes—Comment on the extent to which PPI influenced the study overall. Describe positive and negative effects | Page 12-27 |
| 5: Reflections/critical perspective | Comment critically on the study, reflecting on the things that went well and those that did not, so others can learn from this experience | Page 23-27 |

## **Additional file 2: Table reporting the 21 research studies evaluating the impact of patient and public involvement (PPI) on research, including doctoral research**

| **Author and year** | **Study title** | **Aim** | **Participants** | **Illustrative extracts** |
| --- | --- | --- | --- | --- |
| Beresford CJ, et al. 2024 | Embedding Public Involvement in a PhD Research Project with People Affected by Advanced Liver Disease | The aim of this research was to develop a comprehensive understanding of the care experiences and perspectives of individuals with decompensated advanced liver disease, their carers and the professionals involved. | Five individuals with liver disease and three carers of individuals with liver disease. | - It was useful to speak [PPI members] about their experiences because it encouraged reflection on some of the issues important to them, such as how they access services, and that some aspects of care seem disjointed.  - Changes were made to the participant information leaflet as suggested by members.  - It was great to meet people with different experiences and to see how [PPI members] received the research.  - The discussion … influenced the researcher's thinking around issues of stigma, communication and honesty.  - Being involved in the liver disease project is a personal choice for me to actively contribute to community issues as well as share my life experiences, bring in cultural diversity and gain a sense of empathy for others on a similar journey. [PPI member]  - By contributing to the project, I gain insights into others' attitudes and experiences and also offer support and assistance where possible. [PPI member] |
| Coupe N, Mathieson A. 2019 | Patient and public involvement in doctoral research: impact, resources and recommendations. | The aim of this paper was to explore the impact of PPI on two health‐related doctoral research studies and identify how PPI could be used meaningfully at this level. | Members from university-based PPI panel, service users, staff running the service, three carers from the study area | - Patient information sheets/consent forms – improved readability, ensuring sheets were appropriately informative by identifying and removing unnecessary information and jargon, where possible. - Wording of sensitive topic guide questions and study title – ‘low socioeconomic status area’ was simply change to the ‘[target city] name’ . - Offering potential solutions when faced with recruitment problems, such as gaining access to, and recruiting additional sites, when a site withdrew from the study. - Developing emerging themes, by drawing on their experience, for example the categorization of family carers by community nurses and the interactional work which may have an impact on whether or not the booklet is delivered. - I've enjoyed sharing with you [the researcher], sharing information, thoughts, plans, all the sharing things I've enjoyed. I've enjoyed caring about you and what's happened to you, [laugh] and your disappointments, and the stress. - This was also enjoyable for the researcher, who found the meetings a source of support, in addition to meeting with supervisors improved the researcher's understanding of the availability and workload of community nurses, particularly family carers’ interactions with District Nurses and potential implementers of the booklet. - The researcher considered PPI particularly useful given they were not from the same socio‐economic background as the target population, specifically in reducing potential preconceptions about the factors involved and which elements of the booklet may be challenging. - Layout and content of the booklet in terms of usability & appeal – making it more obvious that only one goal should be selected, choice of food images, making goals colour coordinated for ease of navigation. This was particularly important given the issues around engagement in this population, and the language and literacy barriers identified in the initial qualitative study. |
| Cox R, et al. 2023 | 'Learning and growing together': exploring consumer partnerships in a PhD, an ethnographic study | Describe and evaluate consumer partnerships in a program of doctoral research from the perspective of the consumer co-researchers, the PhD candidate, and the academic supervisors. | The study consisted of seven people with previous experience or expertise in providing healthcare, two people living with disabling conditions, and four relatives and one carer of people with significant healthcare needs. | “…we're learning and growing together. …” [doctoral student]  “…having the two [PPI members] at my PhD confirmation seminar. I felt really supported and valued.” [doctoral student]  “I know that I'm learning stuff as well… the stuff that we're reading, but also in learning about this process of truly trying to engage consumers in the research process… that it's a little bit slower, but actually, you get a lot more out of it in the end.” [doctoral student]  “I personally made a huge contribution, I felt, to those themes, and I was quite chuffed… to see them there in print.” [PPI member] |
| Curry J, et al. 2022 | Developing and testing the Exercise Guide UK website for people with lung cancer: reflections on the added value of patient and public involvement within a doctoral degree. | Discussion of the influence of PPI on a study into digitally delivered physical activity for people diagnosed with lung cancer. | Five PPI members and seven study participants who were living with lung cancer, carers or a family member | The PPI group did not feel this phrase was clear. Therefore, the statement was revised to read, This personally tailored website is for those who have received a lung cancer diagnosis and aims to help you to become more active in a fun and educational way!. The name, Library, was unpopular with those who took part in the qualitative interviews, and the PPI group decided ‘Extra Information’ was more understandable.  For me, Involve [Involve Hull] has just opened my eyes up to a whole world that I didn't realise was out there [research and PPI]…it has opened my eyes up so much. To feel you can make a contribution is very energising – PPI Member 1 It has been very interesting to see how much goes into these sorts of PPI activities. I had no idea it was governed by so many rules, regulations, protocols and things – PPI Member 2 It benefits me in realising I was not the only person with this [lung cancer], which you often feel like you are. So, it was interesting to meet others and hear their experiences – PPI Member 2 For me, the good feeling about the fact that you can be involved in it, but for me, personally, like [PPI member 2] said… y'know realising some people with lung cancer can have a good life with it, some quality of life, sometimes they can enjoy together, which we didn't have. – PPI Member 3 the benefits were...the personal learning and development of the doctoral student. |
| Dawson S, et al. 2020 | Patient and public involvement in doctoral research: Reflections and experiences of the PPI contributors and researcher. Research Involvement and Engagement. | Descriptive account of the PPI process, personal reflections of both the PPI contributors and the researcher in a study exploring the experiences of people from ethnic minority backgrounds in research. | Two PPI members from ethnic minority groups | - This session and discussions with them also enabled Shoba [PhD student] to validate the findings to some extent and think about other topic areas that required further attention. - She asked for our feedback and it was then incorporated, this process also helped Shoba [PhD student] to gain confidence in interviewing study participants. - Working with PPI contributors throughout the project allowed for identifying some problems (e.g. redefining INVOLVE’s definition) which otherwise would have been unanticipated and develop solutions to overcome these barriers, confirm and validate that right decision have been made and increasing researchers’ confidence in those decisions. - Based on their suggestion, a poster was taken to advertise in a local Asian supermarket. This opportunity helped in identifying an individual who attended a local community group and was an active member of the community. They initially volunteered to participate in the study and then supported SD to gain access to the local community groups. - It enabled the researcher to take measures that are culturally appropriate to encourage and facilitate engagement from female participants of Pakistani origin for and during the dissemination event; for example, providing a separate women’s only table(s) with female interpreters. - I [PPI member] enjoyed reading case studies, facilitating the event and the whole process; we had dynamic meetings where we bounced off ideas and we often overstayed our meetings. - It was fascinating that our [PPI members] responses could be identified as common issues for different minority groups, including eastern European, and groups marginalised through mental health issues, homelessness and socio-economic factors. - As the community group members had an established relationship with this individual [PPI member], it enabled the researcher to engage with them, which otherwise would not have been possible. |
| Ditton A, et al. 2025 | A multi-person reflection on using public and patient involvement and engagement to develop dementia research. | Document the process used to gain the expertise of a person living with dementia, highlight some of their challenges and benefits of using PPIE in this way, provide practical tips to improve PPI practices within dementia research. | One person living with early onset dementia and one person retired from a social care job. | - [The doctoral student’s] conversations with [PPI members] made them reflect on their own understanding of dementia and the societal stigmas that still exist about it.  - By listening to [PPI member explain his experiences, reflecting on broader ethical dilemmas within dementia research, and sharing his thoughts, the PPIE process gave [the doctoral student] a fresh perspective on dementia, risk [management], and ethical morality.  - For [the doctoral student], taking part in this PPIE process has improved their comfort and skills communicating with people living with dementia.  - From working together with [the doctoral student], [the PPI members] can now make each other aware of conferences and other events that either [the PPI members, or the doctoral student] are speaking at to help extend [their] dementia network and spread [their] dementia experiences to more audiences which is very important to [them].  - By including [the PPI members] in the research design process [the doctoral student] got the unique insider perspectives of someone living with dementia and somebody living with experiences of supporting somebody with dementia which improved the quality of [their] design, conduct, and resources  - For [a PPI member], it was reassuring to be involved in research that [they] felt was relevant to dementia, because it meant that conversations were being had with people with dementia and not about [them].  - [A PPI member] felt proud to be involved as a PPIE collaborator because since [their] diagnosis many parts of [their] life have been taken away and [their] world made smaller.  - Being included and involved keeps [the PPI member] well, and [they] suspect others might feel a similar sense of purpose and meaning by being asked to contribute to PPIE as well. |
| Doughty J, et al. 2024 | Embedding patient and public involvement into a doctoral study: developing a point-of-care HIV testing intervention for dental settings. | To describe how patient and public involvement (PPI) was embedded in a doctoral study that explored the feasibility of HIV testing in dental settings. | PPI contributors included: (1) three people who regularly attended the dentist who did not have a known diagnosis of HIV, (2) three people who had lived experience of homelessness and had accessed homelessness-specific dental services, and (3) four people PWH (Person with HIV). | - PPI contributed to the redesign of study materials for improved clarity and relevance (e.g., changing “reactive” to “preliminary positive”).  - Study was one of the first to report the views of people with HIV about their attitude towards HIV testing in dental settings.  - Focus on normalising HIV testing in dental settings rather than empowering patients to test. - Posters redesigned from depicting empowered individuals to showing ordinary dental interactions. - PPI helped in training dental professionals and co-facilitated focus groups. |
| Gordon L, et al. 2017 | A research note on the benefit of patient and public involvement in research: the experience of prostate cancer patients regarding information in radiotherapy | Explore the inclusion of patient and public involvement (PPI) in a qualitative study on the experiences of men with prostate cancer regarding information in radiotherapy. | A core group of three men living with prostate cancer. | - There was very strong opinion in the reference group that wives/partners should be included, with one member stating that it would be “no good asking me anything about [my initial consultation] but my wife knows everything, so to get the fullest picture she would need to be interviewed too”.  - The men in the Acorn reference group felt that the gender of an interviewer was not as important as being confident that the interviewer had a knowledge and understanding of cancer and its management. |
| Hansen A, et al. 2024 | Conducting mixed methods research with women in a forensic mental health setting: a reflexive account from a nurse as researcher. | To describe a reflexive account of a mental health nurse undertaking a mixed-methods doctoral study in a secure forensic hospital with women. | Women in a secure forensic hospital, mental health nurses, and the primary author as a nurse-researcher | -Clear communication and positionality statements were used to help participants better understand the researcher’s role. -Adapted data collection methods to respect participant autonomy, allowing breaks and encouraging participant-led strategies -Utilised a reflective diary to manage role conflicts and improve interview techniques. -Balanced personal biases with participant experiences through continuous self-reflection. -Maintained reflexivity throughout all stages of the research process to minimise bias. |
| Hensman-Crook A, et al. 2024 | What matters to you? Public and patient involvement in the design stage of research | The aim of this paper was to explore the impact of public and patient involvement in the co-production of a doctoral research project in the design phase of research conducted in an ethically conscious manner. | One group with six members experienced in involvement in health science research, and one group with five members with no health science experience. | - Both [PPI] groups requested that each chapter of the doctoral thesis has a plain English summary at the beginning of the chapter, with one group adding that the summaries are combined into a final plain English report of the thesis for dissemination.  - Engagement with PPI as outlined above has since led to many changes to the doctoral research moving forward that include: The ontological stance of the research, identification of some main themes to run as a thread throughout the research, development of content for an international scoping review, identification of the best method for data collection for patient research, accountability of the researcher to write a plain English summary at the beginning of each thesis chapter, and a summary report at the end for dissemination for public review. |
| Manikandan M, et al. 2023 | Public and patient involvement in doctoral research during the covid-19 pandemic: Reflections on the process, challenges, impact and experiences from the perspectives of adults with cerebral palsy and the doctoral researcher. | Describing the PPI process and its impact at various stages of the doctoral research process and reflects on the experiences from the perspective of the doctoral researcher and adults with cerebral palsy. | Five adults with cerebral palsy were consulted throughout the doctoral research programme. | - Helped to develop study objectives and informed the statistical analysis plan - Information about the study was clear from the start. - Clarity on inclusion criteria for participant recruitment in the design phase of the study. - Developing plain english or easy read study documentation resulted in information about the study being clear and appealing to potential participants - An accessible website including the contributors’ video comments helped us to recruit people. - Adaptations made this study accessible to people with cerebral palsy who had additional impairments. - This wide dissemination helped us to recruit 43 participants including adults with cerebral palsy, support people and service providers in this study. - Identified factors of importance resulting in research findings that were meaningful and relevant to adults with cerebral palsy. - Helped with the discussion section of the component studies. - Helped identify key target audiences and organizations to share findings with so that the work reaches the target audiences and translates to change in policy and practice. - My experience of being involved in this research was overall very positive. I felt happy to give my time as I knew that it was appreciated and valued. - The doctoral programme provided funding support that helped to reimburse through vouchers, arrange taxi, transport or refreshments for meetings. The PPI contributors equally appreciated the support in valuing their time and commitment. |
| McParland C, et al. 2023 | Involving patients and the public in nursing PhD projects: practical guidance, potential benefits and points to consider | Discuss the nursing research group’s experience of involving patients and the public in PhD research, reflect on some of the benefits gained, and highlight some important considerations for those planning to involve patients and the public in their doctoral research projects. | 12 people with lived experience of two or more chronic conditions. 3 people with lived experience of life-limiting conditions. 9 people with lived experience of heart failure, health professionals working with heart failure or belonging to a targeted cultural group. 5 people with lived experience of cancer, or nurse working with people with cancer. 2 people who use internet for self-diagnosis. | - [PPI members] often helped us to make patient documentation more comprehensible, including information sheets and consent forms.  - [PPI members] supported the research team by acting as ‘cultural brokers’. Discussions with the contributors led [doctoral student] and the research team to rephrase the types of questions he was asking, so that he was approaching the topic of palliative and end of life care more obliquely, using hypothetical scenarios.  - [PPI members] were often involved in producing reports, including manuscripts for submission to academic journals.  - Our group’s [PPI members] could often identify gaps in how we contextualised our research or challenged our assumptions. |
| Pearson H, et al. 2024 | Integration of patient and public involvement in a doctoral research study using the research cycle | This paper aimed to contribute to understanding of how PPI can be effectively incorporated by doctoral students into their research studies. | Four parents (two mothers, two fathers) and one grandparent all of whom were bereaved between 6 months and 10 years at the outset of the first core study | - Ethics amendment for parents to self-refer to the study  - Wording changed to be neutral and non-judgemental list of prompts devised if needed in interviews  - Reviewed coded data for interpretation to discuss researcher’s thinking and approach to analysis discussed components of themes and grouping of codes to provide a narrative of parent treatment decision-making  - Reviewed findings from parent interviews identifying theme names which could be subjective and cause distress to parents  - Highlighted a need to support parents in making treatment decisions for their child |
| Pierce M, et al. 2023 | Embedding formal and experiential public and patient involvement training in a structured PhD programme: process and impact evaluation | Evaluate how formal and experiential PPI training were embedded in a structured doctoral programme and the impact of this training on the individual PhD projects and the overall programme. | 20 participants—eight PPI contributors, four PhD scholars and eight PhD supervisors | - Confirmation that research is worthwhile and relevant  -  Changes to phrasing of information for potential trial participants with respect to reasons for referral for intervention  - Greater clarity to protocol  - Boosted confidence in research project  - Positive ethical approval outcome, likely in part due to the contribution of PPI panel to recruitment materials  - Highlighted areas for future consideration, intervention development, research, and evaluation |
| Simons G, et al. 2023 | The student patient alliance: development and formative evaluation of an initiative to support collaborations between patient and public involvement partners and doctoral students. | Share experiences and reflect on the findings of the evaluation to identify barriers and facilitators to effective partnerships between doctoral students and PPI partners, which could be considered in future initiatives of this kind. | Seven PPI partners from Rheumatology Research Patient Partnership (all of whom were patients with rheumatic and musculoskeletal diseases). | - “It kept my focus on the bench-to-bedside aspect … And it does bring my motivation up quite a bit, particularly if things aren’t going terribly well in the labs or if I’m in a transition part of the project.” [doctoral student]  - I could see, yes you were getting somewhere with it and there’s almost a feeling of excitement that when we next meet up in the next couple of months or so - I’m interested to see, has that line of research gone further. [PPI member]  - I feel more confident speaking about immunology, especially to someone with less/no experience in the field. [doctoral student] |
| Tanay MA, et al. 2022 | Co-designing a cancer care intervention: reflections of participants and a doctoral researcher on roles and contributions | Explore how participants in an Experience-Based Co-Design (EBCD) project perceived their role and involvement in co-designing a cancer care intervention over time | 10 participants: 4 clinicians, 4 PPI group members/patient participants, 1 doctoral researcher, and 1 visual illustrator | - Participants highlighted how their understanding of the co-design process grew over time as they went through the stages that ‘allowed everyone to be heard, their experiences captured and commented on before moving on.’ - Patients felt particularly valued when they worked in the smaller co-design groups, where they were able to contribute to the development of patient information resources - Some clinicians reflected that the experience changed their clinical practice, improving how they provide patient information and support in their daily roles. |
| Tanay MA, et al. 2023 | Patient and public involvement in research: reflections and experiences of doctoral cancer nurse researchers in Europe | The overall aim of the study was to explore the knowledge and experiences of using PPI in research among doctoral students who are registered nurses. | There were ten participants from seven countries and were at various stages of their doctoral study. | - It was a humbling but invigorating experience and marked the moment when patient involvement in my PhD research transitioned from an abstract ideal to a persistent ambition. - However, without meaningful engagement with what constitutes PPI through research that is conducted ‘with’ or ‘by’ patients during the research process, the representation of patients, regardless of the intent, may be insubstantial - This session (on PPI) showed me that as a researcher, I did not reflect this on the academic process especially in identifying needs and planning the research - My research ideas came from talking to patients - As a small research team, we spent time contemplating some of the vital study concepts, and when we piloted our information with patients, after only 5 minutes, our participants were able to identify gaps and misunderstandings within our work - Patient involvement provided an opportunity for patients to assess if the survey content was clear, appropriate, and relevant to cancer survivors. the carers pointed out that the information sheet was too long to read. Instead, they suggested that I shorten the information sheet and indicated which important information should be included in it. - Most of the funding bodies in the United Kingdom request for information about how patients and the public were involved in the planning and conduct of research |
| Teodorowski P, et al. 2024 | Evaluation of public involvement in doctoral research using a four-dimensional theoretical framework. | Contribute to the literature by evaluating public involvement in doctoral research during the COVID‐19 pandemic. | Two public advisors recruited through the Public Adviser Forum at NIHR ARC NWC. | - Clarified the focus of the review and inclusion criteria through the development of a logic model.  - Ensured appropriate inclusion of papers in relation to the review aims.  - Ensuring relevance to members of the public and how results relate to their experience as public advisors  - Recruitment of participants for each group (Polish and South Asian participants' recruitment targets were met).  - Involvement shaped the results and the priorities for the discussion section. |
| Tomlinson J, et al. 2019 | Patient and public involvement in designing and conducting doctoral research: The whys and the hows. | This paper aims to describe and discuss in detail the approaches used by four doctoral researchers to incorporate PPI at different stages of their research studiesand to inform other doctoral researchers about the challenges and limitations relating to PPI that we faced. | In total, at least forty public members were involved including former or current patients. | - JT [PhD student] involved her advisory group in a co-design process that allowed them to generate ideas and create something that could be used by her study population.. - After two weeks of the initial PIS [participant information sheets] being available online, responses were obtained from all panel members, who offered comments which led to changes to the research process, the timescale for the start of the study and the language used within the documents.  - JT [PhD student] developed a draft of the notebook based on these ideas and there were four iterative rounds of feedback and re-design with the advisory group. - Once finalised, a member took the notebook to their luncheon group and asked their peers (average age 83 years) to review the design, layout and language used to ensure that it was appropriate. - The group was familiar with the study through the development of the PIS and consent form and was therefore deemed well-equipped to identify eligible patients. - They distributed study information and approached eligible participants from within their patient support network. - PPI members were able to recruit harder to reach individuals (e.g. age and taking a certain medicine) into the study. - KM [PhD student] found that developing her recruitment strategy with the PPI advisory group was useful as they had wide links with local community groups, e.g. church groups and charity groups, which provided additional resources for consideration. - KM [PhD student] has involved the PPI group to review her literature review narrative synthesis results to ensure its credibility. - The group were able to add their alternative perspectives and provided insightful interpretations of the data. |
| Troya MI, et al. 2019 | Patient and public involvement and engagement in a doctoral research project exploring self‐harm in older adults. | Critically reflect on the process, potential impact and identify challenges and opportunities in involving robust PPIE in a doctoral study. | The PPI was conducted with the Research User Group (RUG) in Keele which consists of 68 lay people. | - Collectively the decision to use NICE [National Institute for Health and Care Excellence] guidelines (CG13) definition for self‐ harm was made, highlighting the possibility of self‐harm with and without suicidal intent - The word dissemination was removed from the information sheet as it was not lay friendly - The group commented on the order of items for discussion in the interview guide, suggesting starting off by asking demographic and clinical questions prior addressing motivations of self‐harm. As well, members suggestion of including triggers to self‐harm was incorporated - Agreed avenues of recruitment: self‐harm support groups, age support groups, female support groups, advertisement in local community, social media - Identification of gaps in the literature: alternative methods of self‐harm (eg overeating) - Suggestion of IT [PhD student] attending self‐harm support groups regularly so potential participants can feel more comfortable in being part of study. This reiterated discussions amongst the research team and resulted in increased participation - Identification of avenues to disseminate findings: GP practices, libraries, retirement homes and third‐sector services - The PPI group provided tips for encountering challenging situations in recruitment: (a) ensuring to state clearly from the start the age range and self‐harm definition used as eligibility criteria; (b) discuss with support workers any difficulties encountered with participants wanting to engage in the study that were not eligible  - The group confirmed appropriateness of additional follow‐up interviews with older adults, as they considered this would aid rapport building and trust between researcher and study participants. - The input to the analysis contributed lay perspective to inter-analyst consensus/triangulation of the data, increasing the potential relevance for older adults who self‐harm. |
| Youssef E, et al. 2022 | Enhancing pharmacy practice doctoral research with patient and public involvement. | The aim of this article is to inspire patient and public involvement (PPI) in pharmacy doctoral candidates projects. | For this project, there were two PPI contributors from a pool of PPI contributors with experience involving pharmacy practice research. | - Infographic of results changed by doctoral candidate after discussion with PPI contributors. - Quotes mapped to different domain by doctoral candidate after discussion with PPI contributors. - Discussion with PPI contributors led to greater contextualisation of literature identified patient barriers to implementation. - Strengthened ethics application. Ethics committee specifically asked about PPI contributor involvement in production of study materials. - Significant changes made to plain English summary, patient participant information sheets, patient participant consent forms and topic guide. |

## **Additional file 3:** **EMBED-Care Guideline for Reporting and Evaluating Impact of Patient and Public Involvement in Doctoral Research**

**EMBED-Care Patient and Public Involvement (PPI) Reflection Guideline**

This guideline is designed to support the process of reflection on the impact of PPI in research. The aim of this guideline is to support capture of relevant information and to encourage a detailed reflection.

**Background Information**

The following should be included to clarify the source of the reflection:

1. The role of the person e.g. researcher or public member.
2. For researchers, the level of study e.g. undergraduate, MPhil, PhD.
3. The years of experience in patient and public involvement.
4. The relation to the research topic.

**Meeting Context**

The following should be included to clarify the context of the meeting:

1. Title of research.
2. The stage of research, for example:

- Research Question Development
- Methodology Development
- Data Collection
- Data Analysis
- Writing-up
- Dissemination

1. Purpose of the meeting.

Both **positive** and **negative** impacts from the PPI meeting should be reflected upon. The following themes may be used to support the process of reflection.

| **Themes** | **Description** |
| --- | --- |
| Research agenda | The topic, research question, initiating projects and funding decisions |
| Research design and delivery | Influencing the research design, tools and choice of method, recruitment, data collection and analysis, writing-up and dissemination |
| Research ethics | The consent process and developing ethically acceptable research |
| Public involved | Acquiring new skills and knowledge, personal development, support and friendship, enjoyment and satisfaction, being rewarded financially |
| Researchers | Better knowledge, enjoyment and satisfaction, challenges to beliefs and attitudes |
| Research participants | A better research process, helping people to feel more at ease in interviews, providing emotional support, providing access to information and services, offering hope and inspiration |
| Wider community | Create trust and acceptance of the research, keep projects grounded and focused on benefits for the community, improve relationships between the community and professionals |
| Community organisations | Gaining credibility, increasing their knowledge, public recognition, positive contribution, link between mainstream systems, developed new alliances |
| Implementation and change | New services, changes in practice, developing capacity for change |
